# Global HIV/AIDS Clinical and Translational Pharmacology 

**DOI:** 10.1155/2012/973627

**Published:** 2012-07-18

**Authors:** Gene D. Morse, Gary Maartens, Charles Chiedza Maponga, Qing Ma

**Affiliations:** ^1^Department of Pharmacy Practice, School of Pharmacy and Pharmaceutical Sciences, University at Buffalo, Buffalo, NY 14260, USA; ^2^Translational Pharmacology Research Core, New York State Center of Excellence in Bioinformatics and Life Sciences, 701 Ellicot Street, Buffalo, NY 14302, USA; ^3^Department of Pharmacology and Physiology, Center for Human Experimental Therapeutics, Clinical and Translational Sciences Institute, University of Rochester School of Medicine and Dentistry, Rochester, NY 14642, USA; ^4^Division of Clinical Pharmacology, Department of Medicine, University of Cape Town, Rondebosch 7701, South Africa; ^5^School of Pharmacy, College of Health Sciences, University of Zimbabwe, Harare, Zimbabwe

The continued development of diagnostic and therapeutic advances for HIV has transformed this once rapidly fatal infection into a chronic disease with an extended lifespan. Initially these benefits were only available to patients in resource-rich countries (RRCs), but since 2003 there has been a global effort through donors and access pricing to provide antiretroviral access to the millions of HIV-infected individuals in resource-limited countries (RLCs) [1–4]. Current combination antiretroviral therapy (cART) achieves viral suppression in a majority of adherent patients [5]. Viral load tests with greater sensitivity have led our appreciation of continued low-level viremia in apparently “suppressed” patients cART while assays that detect latent tissue reservoirs with integrated viral DNA have provided new insight into treatment goals, the source of viral rebound when treatment is interrupted, and the lack of further benefit from treatment intensification [6–11]. A sustained chronic inflammatory state thought to arise in part from translocation of gut microbial products, even during suppressive cART, has led to new mechanistic understanding of chronic ART effects on end organs [12, 13]. These advances have led to new research efforts to augment suppression with pharmacologic strategies to purge the latent viral reservoirs and reduce translocation of intestinal microbial products, to reduce end-organ disease including cardiovascular, neurologic, renal, and bone. The presence of opportunistic infections such as tuberculosis and coinfections including malaria and viral hepatitis (HCV, HBV), as well as cancer, creates additional diagnostic and therapeutic challenges [14].

Clinical and translational pharmacology researchers have made important contributions to our understanding of the complex relationship between antiretroviral pharmacokinetics, pharmacodynamics, and pharmacogenomics [15–27]. As cART regimens are optimized their use in patients with comorbidities is complicated by pharmacologic challenges that include maintaining medication adherence, preventing and managing drug-drug interactions, identifying optimal doses for malnourished patients, drug toxicity monitoring, and pharmacogenomic testing. Each of these areas contributes to the goal of maximizing ART exposure while minimizing risk factors that lead to complications of chronic cART and concurrent medication use. As a result of efforts to treat as many HIV-infected individuals as possible, the requirement for medications for comorbid diseases, the challenge to conducting relevant clinical pharmacology research as quickly as possible is considerable [28]. Ongoing research in the areas of preexposure prevention with oral ART or vaginal or rectal microbicides, and “treatment as prevention” to reduce new HIV infections, along with nanomedicine strategies, pediatric cART dosing, pregnancy, and geriatric considerations also include therapeutic drug monitoring and novel clinical pharmacology components [29–41].

In this special issue, some of these challenges are addressed. With varied global dietary patterns as well as nutritional status, M. Lamorde et al. report on their investigation of “*Effect of food on the steady-state pharmacokinetics of tenofovir, and emtricitabine plus efavirenz in Ugandan adults.”* Other important patient factors are addressed by T. Kakuda et al. in their study of the “*Pharmacokinetics and pharmacodynamics of darunavir and etravirine in HIV-1-infected, treatment-experienced patients in the gender, race, and clinical experience trial.”* T. Mudzviti et al. have examined “*The impact of herbal drug use on adverse drug reaction profiles of patients on antiretroviral therapy in Zimbabwe” *and A. Reda et al. investigated the “*Determinants of adherence to antiretroviral therapy among HIV-infected patients in Africa.” *With regard to coinfections, F. Fehintola et al. studied “*nevirapine-based antiretroviral therapy impacts artesunate and dihydroartemisinin disposition in HIV-infected Nigerian adults.*” W. WirachMaek-a-nantawat et al. have provided a review *of “Challenges in providing treatment and care for viral hepatitis among individuals co-infected with HIV in resource-limited settings.”* Lastly, in a comprehensive review article K. Dooley et al. have reported on “*TB and HIV therapeutics: pharmacology research priorities.”*

In summary, this issue provides some excellent examples of research groups that are leading the way while additional planning and capacity building proceed. The need to conduct clinical pharmacology research is essential and will require expanding research facilities with instrumentation and training for the next generation of researchers in this field. The conduct of this comprehensive research agenda will be facilitated through planning of a global, translational pharmacology research effort that includes academic, industrial, regulatory, and community partnerships in RRCs and RLCs. The need for human and laboratory capacity building integrated with a comprehensive clinical pharmacology quality assurance program is significant and should be further integrated with research planning and implementation on a global scale. This approach is likely to accelerate the use of new treatments in countries that are most impacted by HIV and other infectious diseases within the global community. The Fogarty International Center at the National Institutes of Health in the United States, along with other international research funders like the Wellcome Trust and the European and Developing Countries Clinical Trials Partnership, is training current and future global health scientists. The mechanism for building new research centers with state of the art instrumentation remains a challenge to meeting clinical pharmacology research needs around the world. Development of a plan to address these needs is the focus of an annual workshop at the International AIDS Conference and has provided a forum for discussion, needs assessment, and strategic planning to advance this effort.
